# Pyroptosis-Related LncRNA Signatures Correlate With Lung Adenocarcinoma Prognosis

**DOI:** 10.3389/fonc.2022.850943

**Published:** 2022-03-02

**Authors:** Hua Huang, Zijian Shi, Yongwen Li, Guangsheng Zhu, Chen Chen, Zihe Zhang, Ruifeng Shi, Lianchun Su, Peijun Cao, Zhenhua Pan, Hongbing Zhang, Minghui Liu, Hongyu Liu, Jun Chen

**Affiliations:** ^1^Department of Lung Cancer Surgery, Tianjin Medical University General Hospital, Tianjin, China; ^2^Tianjin Key Laboratory of Lung Cancer Metastasis and Tumor Microenvironment, Tianjin Lung Cancer Institute, Tianjin Medical University General Hospital, Tianjin, China; ^3^Department of Thoracic Surgery, First Affiliated Hospital, School of Medicine, Shihezi University, Shihezi, China; ^4^Quantitative Biomedical Research Center, Department of Population and Data Sciences, University of Texas Southwestern Medical Center, Dallas, TX, United States

**Keywords:** pyroptosis, lncRNA, prognosis, immune cell infiltrating, immune checkpoint

## Abstract

**Background:**

Pyroptosis is a new type of programmed cell death, accompanied by an intense inflammatory response. Previous studies have shown that pyroptosis can modify long-chain non-coding RNA (lncRNA), thereby affecting the occurrence and progression of tumors. However, the underlying role of pyroptosis-related lncRNA in lung adenocarcinoma (LUAD) remains to be elucidated. Therefore, the purpose of our study was to evaluate the prognostic value of pyrolysis-related lncRNA in patients with LUAD.

**Methods:**

A total of 454 LUAD samples were downloaded from The Cancer Genome Atlas (TCGA) database. Pearson’s correlation coefficient was used to identify the pyroptosis-related lncRNAs. Unsupervised consensus clustering was used to identify the various LUAD molecular subtypes. A least absolute shrinkage and selection operator (LASSO) analysis was conducted to construct a prognostic signature.

**Results:**

An 11-lncRNA prognostic signature out of 19 identified pyroptosis-related prognostic lncRNAs was constructed. The patients with LUAD were divided into low-risk and high-risk groups. Patients in the high-risk group had higher score values and mortality. The immune score, stromal score, and estimate score were lower in the high-risk group. The risk score was an independent predictor for OS in multivariate Cox regression analyses (HR > 1, p < 0.01). BTLA, PD-1, PD-L1, CTLA, and CD47 were lower expressed in the high-risk group.

**Conclusions:**

Our study identified an 11-pyroptosis-related lncRNA signature. These findings could further clarify the role of pyroptosis in LUAD and guide the prognosis and individualized treatment of patients.

## Introduction

Lung cancer is the leading cause of cancer-related death (1), great progress has recently been made in the treatment of LUAD, which includes immunological therapy and targeted therapy (2, 3). Nevertheless, people with LUAD still have a low overall survival (OS) rate, with an average 5-year survival of less than 20% (4), and LUAD is the most abundant subtype of lung cancer. The current treatment for LUAD is so limited that the development of effective therapies is urgent. Pyroptosis is a caspase-dependent, pro-inflammatory, programmed cell death, accompanied by the release of a large number of inflammatory factors (5). Both apoptosis and pyroptosis are mediated by caspase. Compared with apoptosis, pyroptosis is a necrotic and inflammatory cell death induced by inflammatory caspase. Given that pyroptosis requires the participation of inflammatory caspase, it can be distinguished from another necrotizing and inflammatory form of programmed cell death —necroptosis and its occurrence does not require the participation of caspase (6). When microorganisms infect host cells exogenously or endogenously, the pattern recognition receptor located in the cytoplasm recognizes and binds to the corresponding ligands through pathogen-associated molecular patterns and damage-related molecular patterns. It forms a multi-protein complex in the cytoplasm, activates inflammatory caspase-1 and caspase-4/5/11, and further cleaves the GSDMD protein to perforate the cell membrane and promote the occurrence of cell pyroptosis. Meanwhile, the inflammasome acts on downstream molecules to promote the activation of inflammatory cytokines, interleukin-1β (IL-1β) and IL-18 and adhesion molecules as well as their release to the outside of the cell through the ruptured cell membrane to recruit and activate more inflammatory cells, amplifying the local and systemic inflammatory response (7). Studies have shown that the activation pathway of pyroptosis is divided into the classic pyroptosis pathway, in which caspase-1 is activated by inflammasomes, and the non-classical pyroptosis pathway, in which caspase-4/5/11 is activated by cytoplasmic lipopolysaccharide. Several reports have recently confirmed that many pyroptosis-related molecules have a significant relationship with tumorigenesis, tumor progression, and tumor therapy. For example, GSDMD is a substrate of inflammatory caspase, which causes pyroptosis by forming small holes in the cell membrane after lysis (8). GSDME, which belongs to the same family as GSDMD, can be activated by caspase-3 when stimulated by chemotherapeutic drugs leading to pyroptosis (9, 10). A previous study had confirmed that the expression of GSDMD in non-small cell lung cancer (NSCLC) tissue is significantly higher, and is related to larger tumor size, more advanced stages, and other more aggressive characteristics (11). It was believed that GSDMD is an independent prognostic marker of LUAD. Further studies have found that GSDMD can inhibit the activation of caspase-3 and polyadenosine ribose polymerase, thereby inhibiting NSCLC cell apoptosis and promoting cancer cell proliferation. On the other hand, knocking out GSDMD can inhibit the epidermal growth factor receptor (EGFR)/AKT pathway and inhibit the proliferation of lung cancer cells (11). A study confirmed that under the action of various small molecule inhibitors against KRAS-, EGFR-, or ALK-driven lung cancer, the intrinsic apoptosis pathway in mitochondria is activated, and activated GSDME mediates apoptosis (12). As mentioned earlier, an increasing number of studies have illustrated the relationship between pyroptosis-related molecules and lung cancer. However, there is a mystery as to the function and underlying mechanism of pyroptosis-related lncRNA in LAUD.

As a subtype of RNA, lncRNAs are more than 200 nucleotides in length (13). They can exert their functions in many biological processes, such as tumorigenesis and apoptosis, by combining with DNA, RNA, or specifically, protein (14, 15). Increasing evidence has shown that lncRNAs are critical factors in the regulation of normal or abnormal cell function status and diseases (16). Dysfunction of lncRNAs can lead to aberrant cell function processes, such as the progression, invasion, and apoptosis of tumor cells, which lead to poor prognoses. In breast cancer, for example, HOX transcript antisense RNA, whose expression level is significantly elevated, has been validated to have an association with the poor prognosis and metastasis (17). Other studies have shown that the abnormal expression of colon cancer-associated transcript 1 runs through the entire disease process of colon cancer occurrence and development, including colon adenoma, colon cancer, colon cancer lymphatic metastasis, and liver metastasis (18). Many lncRNAs perform important functions in lung cancer (19, 20). Metastasis associated with lung adenocarcinoma transcript 1 (MALAT1) is abnormally expressed in tumors of the breast, bladder, liver, and prostate, especially in NSCLC (21–24). Many studies have shown that MALAT1 participates in regulating tumor cell migration, and it can regulate metastasis-related genes at the transcription level or post-transcription level to enhance the migration ability of lung cancer cells (25). Another study had confirmed that MALAT1 plays a role in lung cancer metastasis by regulating the expression of related target genes rather than alternative splicing by establishing a MALAT1 gene knockout model (26). However, the expression pattern and function of lncRNA in LUAD has yet to be systematically analyzed.

Based on the TCGA database, our study used bioinformatics methods to analyze the differential expression of pyroptosis-related lncRNAs in LUAD tissues and normal tissues and employed relevant statistical methods to screen candidates and construct a risk model, aiming to explore its potential predictive value and analyze related biological functions. We hope our research will help elucidate the role of pyroptosis-related lncRNA in LUAD.

## Materials and Methods

### Dataset Acquisition and Processing

The mRNA expression data of LUAD were downloaded from the TCGA database (https://portal.gdc.cancer.gov/repository), and we obtained corresponding clinical data. A total of 454 cases were included in this study, which were randomly separated into a training cohort for pyroptosis-related lncRNA signature construction and a validation cohort for model validation.

### Identification of Pyroptosis-Related Prognostic lncRNAs

A total of 52 pyroptosis-related genes were retrieved from previous research and literature; they are shown in **Supplementary Table S1**. We screened the lncRNAs in the TCGA cohort according to gene annotation and obtained a total of 13,413 lncRNAs. The Pearson correlation coefficient was conducted to evaluate the correlation between 52 pyroptosis-related genes and lncRNAs. The lncRNA with an absolute correlation coefficient >0.5 and a P value < 0.001 was considered as a pyroptosis-related lncRNA, and we screened a total of 1457 pyroptosis-related lncRNAs. Then, a univariate Cox regression analysis of OS was performed to screen pyroptosis-related lncRNAs with prognostic value; P < 0.05 was considered to be related to the prognosis, a total of 19 pyroptosis-related lncRNAs with prognostic value were screened.

### Consensus Clustering

An unsupervised consensus clustering algorithm was applied to classify all patients with LUAD into clusters according to the similarities of the pyroptosis-related lncRNA expression levels by using the “ConsensusClusterPlus” R package. A survival analysis was then conducted to explore the prognosis of various clusters. Immune cell infiltration was compared and analyzed by CIBERSORT and ssGSEA methods.

### Construction and Evaluation of the Prognostic Model

To minimize the risk of overfitting, a LASSO Cox regression analysis was performed to build a prognostic model in the training cohort (27). The LASSO algorithm was used for variable selection and shrinkage with the “glmnet” R package. The independent variable was the normalized expression matrix of 19 pyroptosis-related lncRNAs with prognostic value, and the response variables were OS and status of patients in the training cohort. Penalty parameter (λ) for the model was determined by tenfold cross-validation following the minimum criteria. The risk score of each patient was calculated based on the standardized expression level of lncRNA and its corresponding regression coefficient. The formula was established as follows: score = e^sum (each lncRNA expression × corresponding coefficient)^. We used the median risk score as a cutoff to classify patients with LUAD into low-risk and high-risk groups, and a Kaplan–Meier survival curve was further employed to analyze the difference in survival prognosis employing the “Survival” package. The “survivalROC” package was used to draw the receiver operating characteristic (ROC) curve of the 1-year, 3-year, and 5-year OS rates of LUAD. To validate the predictive power of our model, the same above formula was used for verification in the validation cohort.

### Evaluation of Immune Infiltration

The estimate algorithm was utilized to calculate the immune score, stromal score, and estimate score for each patient with LUAD using the “ESTIMATE” package in the R software. The CIBERSORT analytical tool was adopted to identify the abundance of 22 types of immune cells in various LUAD clusters. Moreover, the enrichment scores of 16 immune cells and 13 immune functions for each LUAD sample were calculated by the "gene set variation analysis (GSVA)" package.

### Function Enrichment Analysis

To explore the possible enrichment pathways among different risk score groups, gene set enrichment analysis (GSEA) and GSVA analysis were applied to elucidate relevant signaling pathways in various groups, and functional enrichment analyses were performed using the "clusterProfiler" package.

### Cell Culture

A human LUAD cell line (A549, NCI-H1975) and a normal lung epithelial cell line (BEAS-2B) were obtained from the American Type Culture Collection (Manassas, VA, USA). All cells were cultured in Roswell Park Memorial Institute-1640 medium supplemented with 10% fetal bovine serum.

### Tissue Samples

We collected 5 pairs of LUAD and paracancerous tissues from surgical patients in the Tianjin Medical University General Hospital (TJMUGH). Samples were stored at −80°C until use. The ethics committee of TJMUGH approved this study.

### RNA Isolation and Quantitative Real-Time Polymerase Chain Reaction

Following the procedure previously described (28), we extracted total RNA from the samples. We synthesized cDNA using a PrimeScript RT Reagent Kit (TaKaRa). Then, cDNA was subjected to quantitative real-time polymerase chain reaction (RT-qPCR) by the ABI 7900HT platform (Applied Biosystems, USA). We used β-actin mRNA as an internal reference to normalize the 11 lncRNAs by the comparative Ct method. The primer sequence involved in this study is shown in **Supplementary Table S2**.

### Statistical Analysis

The “timeROC” package was used to analyze the ROC curve. Univariate and multivariate Cox regression analyses were performed to determine independent prognostic factors for OS. All the statistical analyses of the data are based on the R platform (Version 4.0.2) and GraphPad Prism 8. If there were no special instructions for the above analysis methods, P < 0.05 was considered statistically significant.

## Results

### Identification of Pyroptosis-Related lncRNAs in LUAD

**Figure 1:**
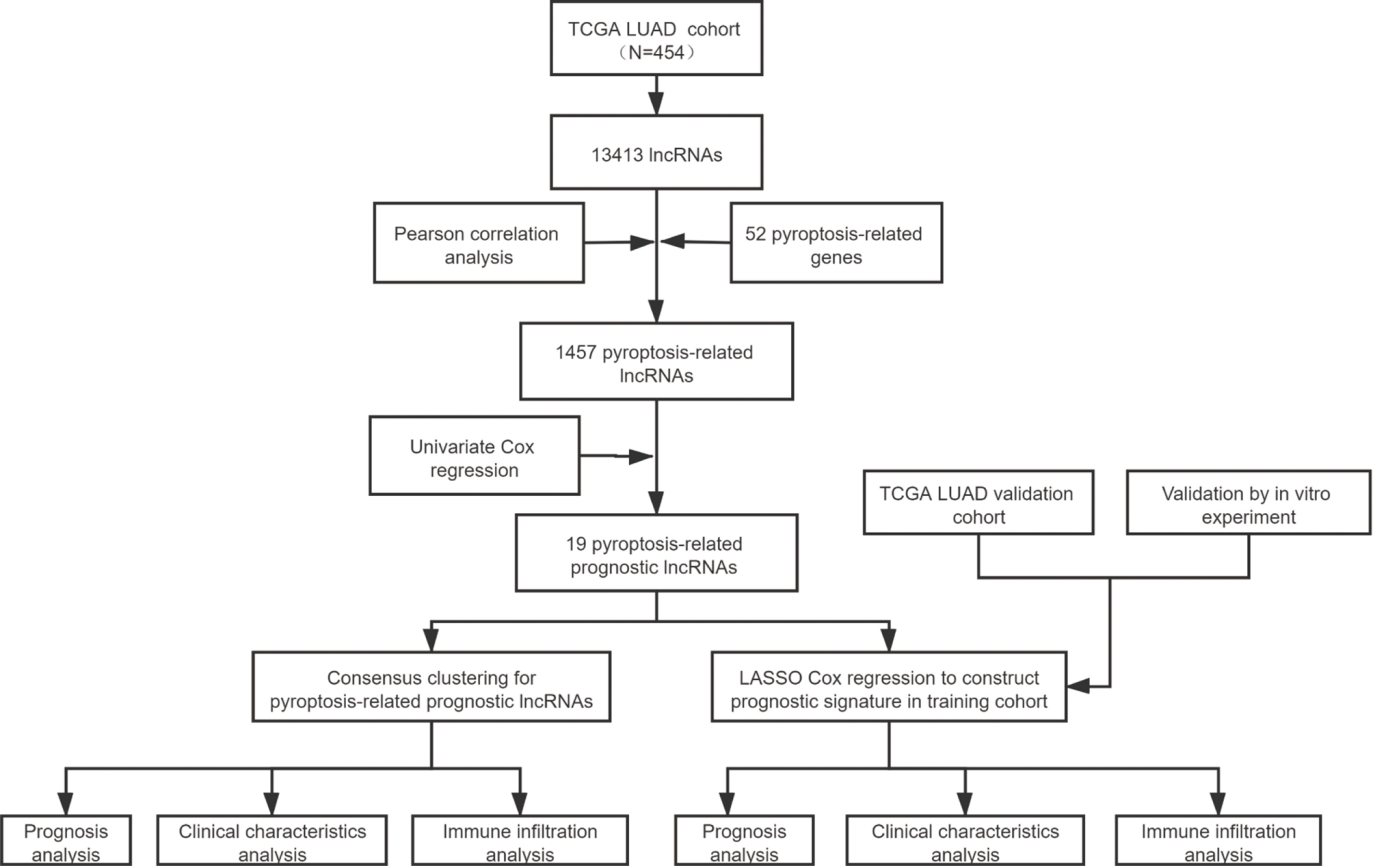
The entire analytical process of the study.

The detailed process is shown in **Figure 1**. A total of 454 patients with LUAD were included in this analysis, and detailed clinical information is shown in **Supplementary Table S3**. A total of 52 pyroptosis-related genes came from previous research and literature. Employing a Pearson correlation analysis, we identified 1457 pyroptosis-related lncRNAs. Then, a univariate regression analysis was performed to identify 19 pyroptosis-related lncRNAs with prognostic value in LUAD.

### Consensus Clustering Categorized Patients According to Pyroptosis-Related Prognostic lncRNAs

**Figure 2:**
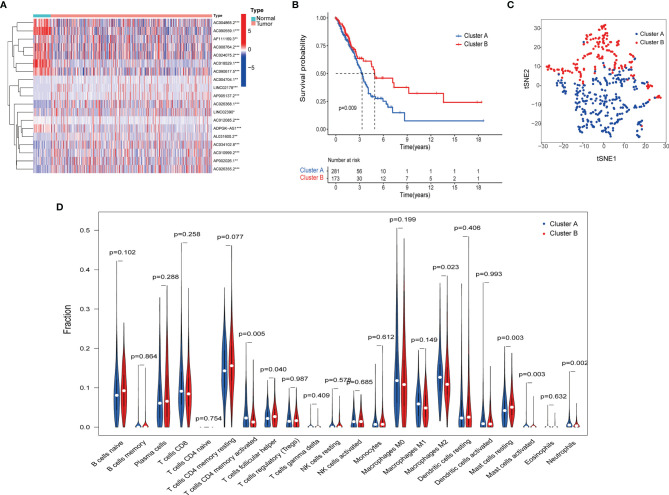
Pyroptosis-related lncRNAs molecular patterns in the TCGA cohort. **(A)** Difference between normal and tumor tissue of 19 LncRNAs related to the prognosis of LUAD. **(B)** Kaplan–Meier method was used to plot the OS curve for the cluster A and B. **(C)** tSNE analysis of cluster A and B. **(D)** The infiltrating levels of 22 immune cell types in cluster A and cluster B.

**Figure 3:**
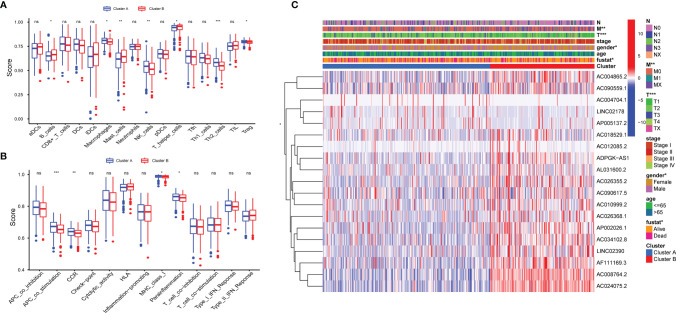
Distinct Clinical characteristics and immune cell infiltrations and function with molecular patterns in LUAD. **(A, B)** The enrichment scores of 16 immune cells **(A)** and 13 immune-related functions **(B)** ;*P < 0.05, **P < 0.01, ***P < 0.001, and ^ns^P > 0.05. **(C)** Heatmap of correlation of the two clusters with clinicopathologic features.

To identify distinct molecular patterns based on the expression of pyroptosis-related lncRNAs with prognostic value, unsupervised consensus clustering was applied to separate patients with LUAD into two clusters, with k=2 found to be optimal clustering stability (**Figures S1A–C**). As shown in **Figure 2A**, significant differences were observed in the expression levels of 19 pyroptosis-related lncRNAs, with a prognostic value between cancer and adjacent cancers. The OS rate of patients with LUAD in cluster A was poorer (**Figure 2B**). Furthermore, t-distributed stochastic neighbor embedding demonstrated that cluster A and cluster B can be completely distinguished (**Figure 2C**). We further evaluated the differences in the tumor immune microenvironment between different molecular patterns. **Figure 2D** shows the infiltration abundance of 22 types of immune cells in each cluster by CIBERSORT analysis. Cluster A had a higher abundance of memory-activated CD4 T cells and M2 macrophages, and a lower abundance of T cells follicular helper compared with cluster B. Moreover, the single-sample GSEA showed significant differences in immune infiltrations and immune functions between cluster A and cluster B. Cluster A had a higher abundance of macrophages, Th2 cells, natural killer cells, antigen-presenting cell co-stimulation, and chemokine receptors, and a lower abundance of B cells and mast cells (**Figures 3A, B**). Comparing the clinicopathological characteristics of various clusters, the expression profiles of pyroptosis-related lncRNAs were significantly different in LUAD (**Figure 3C**).

### Construction and Validation of the Pyroptosis-Related lncRNA Signature

**Figure 4:**
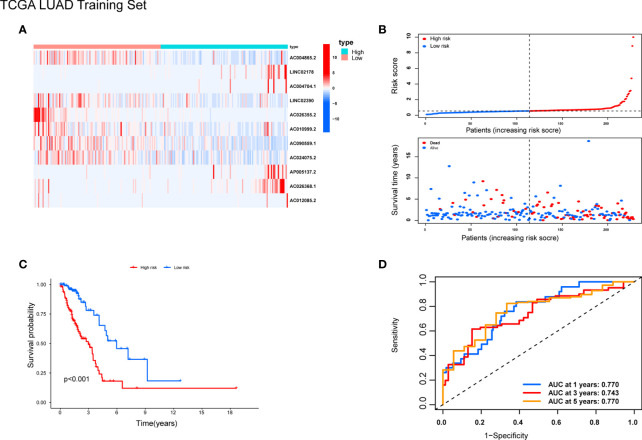
Prognostic analysis of 11-lncRNAs model in the TCGA training cohort. **(A)** Heatmap of 11 pyroptosis-related lncRNAs in the training cohort. **(B)** Distribution of survival time and risk scores. **(C)** Survival analysis in the TCGA training cohort. **(D)** ROCs for 1, 3, and 5 year survival time based on the risk score.

**Figure 5:**
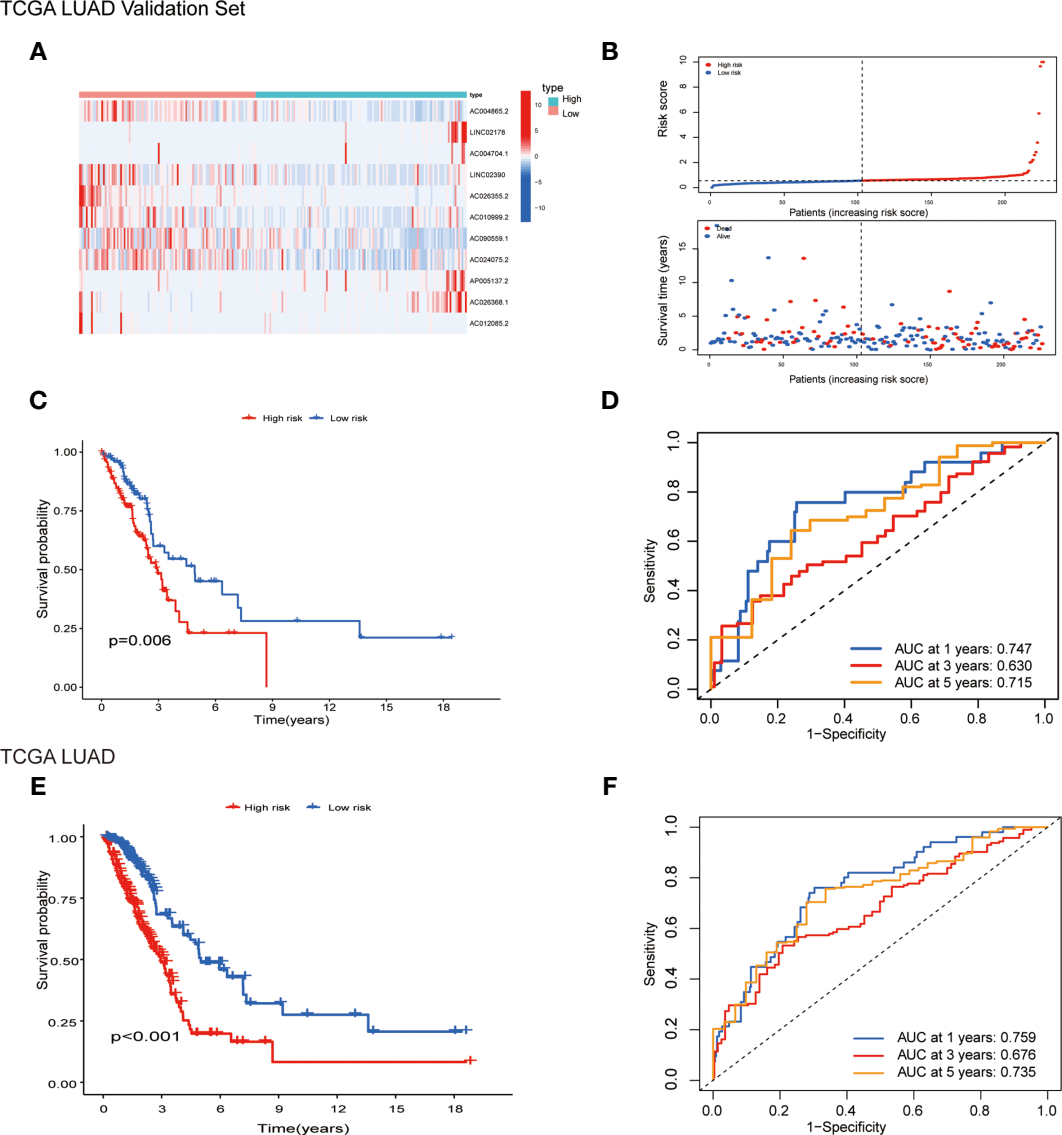
Prognostic analysis of 11-lncRNAs model in the TCGA validation cohort and entire TCGA cohort. **(A)** Heatmap of 11 pyroptosis-related lncRNAs in the validation cohort. **(B)** Distribution of survival time and risk scores. **(C)** Survival analysis in the TCGA validation cohort. **(D)** ROCs for 1, 3 and 5 year survival time based on the risk score. **(E)** Survival analysis in the entire TCGA cohort. **(F)** ROCs for 1, 3 and 5 year survival time based on the risk score.

Patients were randomly separated into training cohorts and validation cohorts. We conducted a LASSO Cox analysis to construct an 11-lncRNA prognostic signature using the 19 identified pyroptosis-related prognostic lncRNAs in the training cohort. **Figures S2A, B** show the coefficient and partial likelihood deviance of the prognostic signature. Patients were separated into high-risk and low-risk groups by the median values in the training cohort. The relative expression levels of the 11-lncRNAs were significantly different in the cancer and adjacent tissues (**Figure 4A**). Patients in the high-risk group had higher score values and mortality (**Figures 4B, C**). We performed R software to draw the time-dependent ROC curve, the results showed that the area under the curve for 1, 3, and 5 years reached 0.770, 0.743, and 0.770, respectively (**Figure 4D**). Similar results have also been verified in the validation cohort, suggesting that our model has a strong prognostic value (**Figures 5A–D**). We also combined the entire TCGA LUAD cohort to verify the stability of this model. The ROC curve further demonstrated that our model has a strong prognostic value (**Figures 5E, F**).

**Figure 6:**
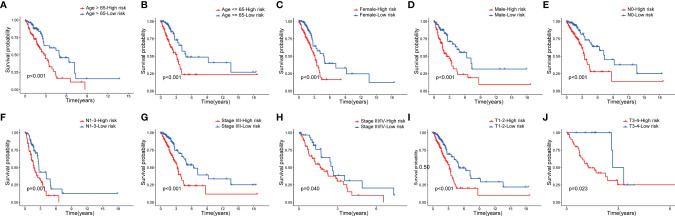
Survival analysis of clinical stratification of OS in the TCGA cohort. **(A, B)** age (< = 65 or > 65 years old). **(C, D)** gender (female or male). **(E, F)** N (N0 or N1-3). **(G, H)** tumor stage (I–II or III-IV). **(I, J)** T (T1-2 or T3-4).

Next, to assess whether the model retains its predictive ability in the subgroups of different clinical features, we verified the prognosis of risk score in various groups of patients with LUAD, which found that the high-risk group had a poor prognosis in patients aged >65, aged ≤65 (**Figures 6A, B**), and by sex subgroup (**Figures 6C, D**). Moreover, the high-risk group had a poor prognosis in patients with LUAD at the N0 stage, N1-3 stage (**Figures 6E, F**), stage I/II, stage III/IV (**Figures 6G, H**), T1-2 stage, and T3-4 stage (**Figures 6I, J**).

### Pathway Enrichment Analysis

**Figure 7:**
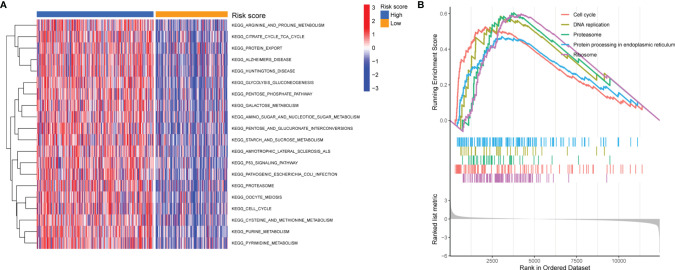
Pathway Enrichment Analysis. **(A, B)** GSVA and GSEA of biological pathways between the high- and low-risk group.

To elucidate the potential biological functions of the 11-lncRNA pyroptosis-related signature, GSVA and GSEA were further performed to investigate the key pathways of various risk groups. As shown in **Figure 7A**, GSVA identified most metabolism-related pathways that were enriched in the high-risk group, including arginine and proline metabolism, the tricarboxylic acid cycle, glycolysis, and gluconeogenesis. The GSEA analysis showed that the high-risk group was significantly enriched in the cell cycle, proteasome, protein processing in the endoplasmic reticulum, DNA replication, and the ribosome (**Figure 7B**).

### Evaluation of Immune Infiltration

**Figure 8:**
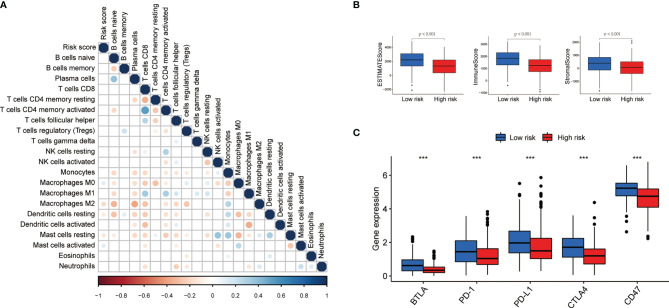
Immune infiltration discrepancy in different risk groups. **(A)** The correlation of the risk scores and immune cells infiltration. **(B, C)** The differences of ESTIMATE scores and expression of five common immune checkpoints in different risk groups ;***P < 0.001.

We investigated the role of pyroptosis-related lncRNAs in the LUAD tumor microenvironment. The CIBERSORT demonstrated that the abundance of M0 macrophages was positively associated with the risk score (**Figure 8A**). We also calculated the immune score, stromal score, and estimate score for each patient by the ESTIMATE algorithm; the results demonstrated that the immune score, stromal score, and estimate score were lower in the high-risk group (**Figure 8B**). To further explore the differences in the response to immunotherapy between the two groups, we compared the differences in the expression of immune checkpoints. As shown in **Figure 8C**, B- and T-lymphocyte attenuator (BTLA), programmed cell death (PD)-1, PD ligand 1 (PD-L1), cytotoxic T lymphocyte-associated antigen (CTLA), and CD47 were all elevated in the low-risk group.

### Independent Prognostic Value of the Risk Score

**Figure 9:**
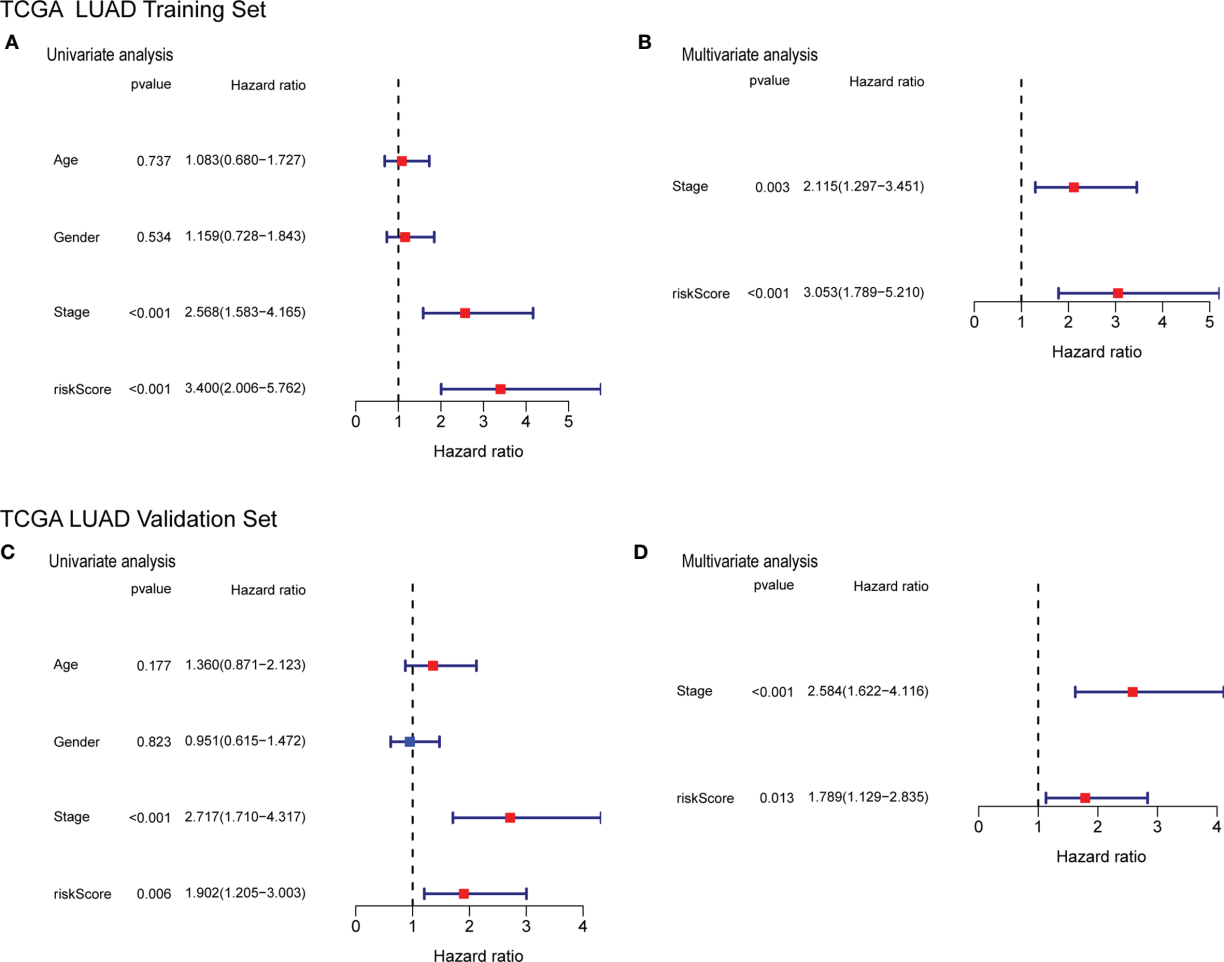
Forrest plot of the univariate and multivariate Cox regression analyses regarding OS in the TCGA training cohort **(A, B)** and the TCGA validation cohort **(C, D)**.

To determine whether the risk score can be used as an independent factor for predicting OS, we performed a univariate Cox regression analysis on clinical parameters and the risk score. The risk score was significantly associated with OS in both the training and the validation cohort (HR= 3.400, 95% CI = 2.006-5.762, P< 0.001; HR= 1.902, 95% CI = 1.205-3.003, P= 0.006, respectively) (**Figures 9A, C**) The multivariate Cox regression analysis revealed that the risk score still had a statistically significant impact on survival and prognosis after adjusting for other confounding factors (Training cohort: HR =3.053, 95% CI = 1.789-5.210, P<0.001; Validation cohort: HR=1.789, 95% CI = 1.129-2.835, P = 0.013) (**Figures 9B, D**).

### The Expression Levels of the 11 Pyroptosis-Related lncRNAs

**Figure 10:**
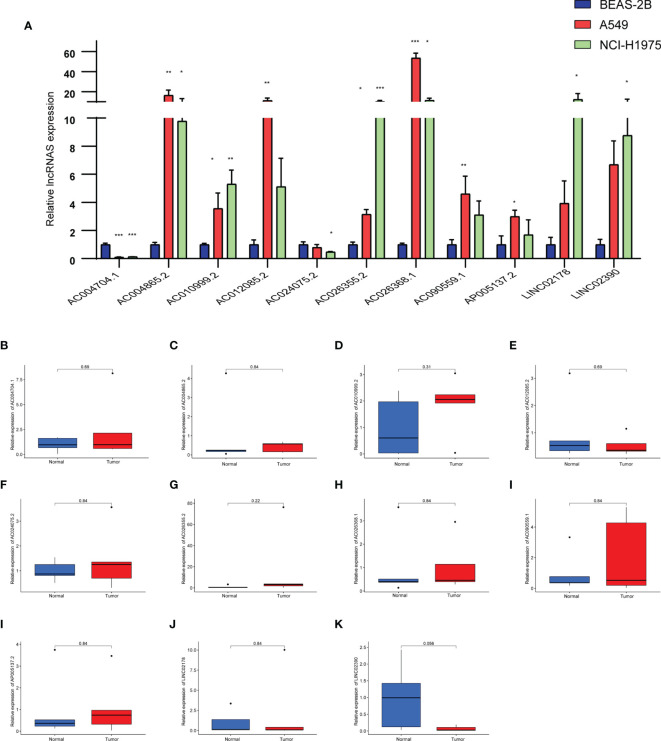
**(A)** Expression levels of 11 pyroptosis-related lncRNAs in the normal lung epithelial cell line BEAS-2B and two LUAD cell lines (A549, NCI-H1975) by RT-qPCR. **(B–K)** Expression levels of 11 pyroptosis-related lncRNAs in LUAD tissues and corresponding normal tissues by RT-qPCR. Data represent the mean ± SD. *P < 0.05, **P < 0.01, ***P < 0.001. P values were determined by one-way ANOVA.

We analyzed the differences of 11 lncRNAs in the normal lung epithelial cell line BEAS-2B and two LUAD cell lines (A549, NCI-H1975) by RT-qPCR. As shown in **Figure 10A**, there were obvious differences in the expression of these lncRNAs, except that the expression levels of AC004704.1 and AC024075.2 were downregulated in the tumor cell lines, whereas the other 9 lncRNAs were upregulated. We further verified the expression level of 11 lncRNAs in 5 pairs of LUAD tissues and adjacent tissues. Due to the small number, however, we did not find any difference between cancer and adjacent cancer (**Figures 10B–K**).

## Discussion

LUAD is the most common NSCLC subtype among non-smokers. Despite recent progress in cancer treatment, the OS rate of LUAD is still disappointing due to the lack of reliable early prognostic indicators. Therefore, it is urgent to find a biomarker closely related to LUAD to guide individualized treatment and accurately predict patient prognoses. In recent years, pyroptosis has received greater attention, and its related molecules have the potential to become biomarkers.

Pyroptosis has been extensively studied in various cancers, and many molecules related to pyroptosis participate in the tumorigenesis and progression of cancer. For example, the expression level of GSDMB in breast cancer cells is higher than that in normal breast tissue and is related to the high metastasis rate and low patient survival rate (29). In a study on the correlation between GSDME methylation and various clinicopathological parameters, with breast cancer as an example, the GSDME promoter methylation value of lobular adenocarcinoma was significantly higher than that of ductal adenocarcinoma (30). This study also observed a significant correlation between GSDME promoter methylation and tumor stage, with the highest degree of methylation in stage III, and the same degree of methylation in stages I and II (30).

Increasing data show that lncRNA abnormalities, such as overexpression, deletion, or mutation, have a driving effect on the malignant biological behavior of tumors, such as tumor formation, progression, metastasis, and recurrence. For example, MALAT1 can promote tumor metastasis chiefly by regulating epithelial-to-mesenchymal transition in NSCLC. Some scholars used miR-101-3p to knock down MALAT1, thereby inhibiting the growth and metastasis of NSCLC *via* the PI3K/AKT signaling pathway (31). One study had indicated that miR-142-3p exerts a tumor suppressor effect in NSCLC by inhibiting the MALAT1/β-catenin signaling pathway. In short, lncRNA participates in many important biological processes of LUAD. Nevertheless, pyroptosis-related lncRNAs in LUAD deserve more attention (32).

In this study, a model of 11 pyroptosis-related lncRNAs in LUAD was constructed with a LASSO Cox regression analysis and other bioinformatics analyses. We constructed a model of 11 pyroptosis-related lncRNAs with prognosis value, and it successfully predicted OS (**Figure 4**). None of the 11 lncRNAs (AC004865.2, AC004704.1, LINC02390, AC0109992, AC024075.2, AP0051372, AC026368.1, AC012085.2, LINC02178, AC026355.2 and AC090559.1) have previously been reported in LUAD and other cancers. They could become potential prognostic markers, and need to be further explored and studied in LUAD.

A similar study established a signature of seven pyroptosis-related lncRNAs to predict the prognosis of patients with LUAD through bioinformatics analysis, which could function as prognostic biomarkers for LUAD (33). The AUCs of the seven pyroptosis-related lncRNAs signature in the training and validation cohorts were respectively 0.757 and 0.728 at 1 year. In contrast, the prediction results of the model in this study at 1 year are 0.770 and 0.747, respectively, indicating that the proposed model is slightly more predictable. In addition, the signature was also more predictive at long-term follow-up, with 5-year AUCs of 0.77 and 0.73 in the training cohort and validation cohort, respectively. (**Figures 4D**, **5D, F**).

We further explored whether the signature we constructed maintained its predictive ability in subgroups with different clinical characteristics. The results show that the model accurately distinguished high- and low-risk groups, regardless of age, sex, and pathological stage (**Figure 6**).

The results of the GSVA and GSEA identified pathways significantly enriched in high-risk groups, including cell cycle, DNA replication, proteasome, protein processing in the endoplasmic reticulum, and ribosome pathways (**Figures 7A, B**). Our results were similar to a previous study reporting that the expression of GSDMD was reduced in gastric cancer tissues, and the reduction in GSDMD expression significantly promoted tumor proliferation *in vivo* and *in vitro*. GSDMD regulates cell cycle-related proteins in gastric cancer through a series of pathways to accelerate S/G2 cell transformation (34). This result implies that pyroptosis could have a protective effect on NSCLC cells, which could be used as a potential diagnosis and treatment strategy.

To explore the influence of pyroptosis-related lncRNAs on the LUAD tumor microenvironment, the proportion of 22 immune cell types were calculated stromal score, immune score, and estimate score for each patient were obtained (**Figure 8**). A recent study confirmed that cytotoxic lymphocytes kill tumor cells *via* pyroptosis. Granzyme A induces pyroptosis by hydrolyzing GSDMB, which results in tumor cell death, indicating that pyroptosis positively affects the tumor immune response process (35). However, the influence of pyroptosis on the tumor microenvironment and immunotherapy is still unclear. The relationship between pyroptosis and immunity needs to be further explored and verified.

We also found that the expression levels of BTLA, PD-1, PD-L1, and CTLA increased in the low-risk group (**Figure 8C**). The discovery of PD-1 and PD-L1 has made their mechanism involving the occurrence and development of tumors an important research topic. Researchers have explored the expression of PD-1 and PD-L1 in a variety of cancers and related immunotherapies. The results show that the model might provide guidance for future immunotherapy.

Our study has limitations. First, our study methods were not comprehensive. Experiments exploring different aspects of molecular biology are necessary to further analyze the mechanism of pyroptosis-related lncRNAs in the tumorigenesis and development of LUAD. Second, the model was only validated in the TCGA validation cohort; thus, it needs to be further externally validated in larger sample sizes and different LUAD cohorts.

In short, an 11-pyroptosis-related lncRNA prognostic signature was constructed, which could function as an independent prognostic variable for patients with LUAD. We hope this model will be useful as a reference to predict patient survival and guide related treatments for patients with LUAD.

## Data Availability Statement

The original contributions presented in the study are included in the article/**Supplementary Material**. Further inquiries can be directed to the corresponding author.

## Author Contributions

HH, ZS, and YL wrote the main manuscript text. GZ, CC, and ZZ prepared Figures. RS, LS, PC, and ZP contributed to data analysis. HZ and ML contributed to data acquisition. HL and JC revised the manuscript. All authors reviewed the manuscript. All authors contributed to the article and approved the submitted version.

## Funding

This work was supported by the National Natural Science Foundation of China (82072595, 82172569, 81773207 and 61973232), Natural Science Foundation of Tianjin (19YFZCSY00040, and 19JCYBJC27000), Shihezi University Oasis Scholars Research Startup Project (LX202002). Project of Tianjin Lung Cancer Institute (TJLCZD2021-04,TJLCZD2021-01,TJLCMS2021-01,TJLCZJ2021-03)Tianjin Key Medical Discipline (Specialty) Construction Project.

## Conflict of Interest

The authors declare that the research was conducted in the absence of any commercial or financial relationships that could be construed as a potential conflict of interest.

## Publisher’s Note

All claims expressed in this article are solely those of the authors and do not necessarily represent those of their affiliated organizations, or those of the publisher, the editors and the reviewers. Any product that may be evaluated in this article, or claim that may be made by its manufacturer, is not guaranteed or endorsed by the publisher.

## References

[1] Bray F Ferlay J Soerjomataram I Siegel RL Torre LA Jemal A. Global Cancer Statistics 2018: GLOBOCAN Estimates of Incidence and Mortality Worldwide for 36 Cancers in 185 Countries. CA Cancer J Clin (2018) 68(6):394–424. doi: 10.3322/caac.21492 30207593

[2] Sui H Ma N Wang Y Li H Liu X Su Y. Anti-PD-1/PD-L1 Therapy for Non-Small-Cell Lung Cancer: Toward Personalized Medicine and Combination Strategies. J Immunol Res (2018) 2018:6984948. doi: 10.1155/2018/6984948 30159341 PMC6109480

[3] Farhat FS Houhou W. Targeted Therapies in non-Small Cell Lung Carcinoma: What Have We Achieved So Far? Ther Adv Med Oncol (2013) 5(4):249–70. doi: 10.1177/1758834013492001 PMC3707340 23858333

[4] Lin JJ Cardarella S Lydon CA Dahlberg SE Jackman DM Janne PA. Five-Year Survival in EGFR-Mutant Metastatic Lung Adenocarcinoma Treated With EGFR-TKIs. J Thorac Oncol (2016) 11(4):556–65. doi: 10.1016/j.jtho.2015.12.103 PMC4979601 26724471

[5] Man SM Karki R Kanneganti TD. Molecular Mechanisms and Functions of Pyroptosis, Inflammatory Caspases and Inflammasomes in Infectious Diseases. Immunol Rev (2017) 277(1):61–75. doi: 10.1111/imr.12534 28462526 PMC5416822

[6] Aachoui Y Sagulenko V Miao EA Stacey KJ. Inflammasome-Mediated Pyroptotic and Apoptotic Cell Death, and Defense Against Infection. Curr Opin Microbiol (2013) 16(3):319–26. doi: 10.1016/j.mib.2013.04.004 PMC3742712 23707339

[7] Jorgensen I Miao EA. Pyroptotic Cell Death Defends Against Intracellular Pathogens. Immunol Rev (2015) 265(1):130–42. doi: 10.1111/imr.12287 PMC4400865 25879289

[8] Shi J Gao W Shao F. Pyroptosis: Gasdermin-Mediated Programmed Necrotic Cell Death. Trends Biochem Sci (2017) 42(4):245–54. doi: 10.1016/j.tibs.2016.10.004 27932073

[9] Wang Y Gao W Shi X Ding J Liu W He H. Chemotherapy Drugs Induce Pyroptosis Through Caspase-3 Cleavage of a Gasdermin. Nature (2017) 547(7661):99–103. doi: 10.1038/nature22393 28459430

[10] Wang Y Yin B Li D Wang G Han X Sun X. GSDME Mediates Caspase-3-Dependent Pyroptosis in Gastric Cancer. Biochem Biophys Res Commun (2018) 495(1):1418–25. doi: 10.1016/j.bbrc.2017.11.156 29183726

[11] Gao J Qiu X Xi G Liu H Zhang F Lv T. Downregulation of GSDMD Attenuates Tumor Proliferation *via* the Intrinsic Mitochondrial Apoptotic Pathway and Inhibition of EGFR/Akt Signaling and Predicts a Good Prognosis in Nonsmall Cell Lung Cancer. Oncol Rep (2018) 40(4):1971–84. doi: 10.3892/or.2018.6634 PMC6111570 30106450

[12] Lu H Zhang S Wu J Chen M Cai MC Fu Y. Molecular Targeted Therapies Elicit Concurrent Apoptotic and GSDME-Dependent Pyroptotic Tumor Cell Death. Clin Cancer Res (2018) 24(23):6066–77. doi: 10.1158/1078-0432.CCR-18-1478 30061362

[13] Noh JH Kim KM McClusky WG Abdelmohsen K Gorospe M. Cytoplasmic Functions of Long Noncoding RNAs. Wiley Interdiscip Rev RNA (2018) 9(3):e1471. doi: 10.1002/wrna.1471 29516680 PMC5963534

[14] Morlando M Fatica A. Alteration of Epigenetic Regulation by Long Noncoding RNAs in Cancer. Int J Mol Sci (2018) 19(2):570. doi: 10.3390/ijms19020570 PMC5855792 29443889

[15] Fatica A Bozzoni I. Long non-Coding RNAs: New Players in Cell Differentiation and Development. Nat Rev Genet (2014) 15(1):7–21. doi: 10.1038/nrg3606 24296535

[16] Slack FJ Chinnaiyan AM. The Role of Non-Coding RNAs in Oncology. Cell (2019) 179(5):1033–55. doi: 10.1016/j.cell.2019.10.017 PMC7347159 31730848

[17] Gupta RA Shah N Wang KC Kim J Horlings HM Wong DJ. Long non-Coding RNA HOTAIR Reprograms Chromatin State to Promote Cancer Metastasis. Nature (2010) 464(7291):1071–6. doi: 10.1038/nature08975 PMC3049919 20393566

[18] Alaiyan B Ilyayev N Stojadinovic A Izadjoo M Roistacher M Pavlov V. Differential Expression of Colon Cancer Associated Transcript1 (CCAT1) Along the Colonic Adenoma-Carcinoma Sequence. BMC Cancer (2013) 13:196. doi: 10.1186/1471-2407-13-196 23594791 PMC3639026

[19] Zhen Q Gao LN Wang RF Chu WW Zhang YX Zhao XJ. LncRNA DANCR Promotes Lung Cancer by Sequestering miR-216a. Cancer Control (2018) 25(1):1073274818769849. doi: 10.1177/1073274818769849 29651883 PMC6852365

[20] Shen QM Wang HY Xu S. LncRNA GHET1 Predicts a Poor Prognosis of the Patients With non-Small Cell Lung Cancer. Eur Rev Med Pharmacol Sci (2018) 22(8):2328–33. doi: 10.26355/eurrev_201804_14823 29762836

[21] Guffanti A Iacono M Pelucchi P Kim N Solda G Croft LJ. A Transcriptional Sketch of a Primary Human Breast Cancer by 454 Deep Sequencing. BMC Genomics (2009) 10:163. doi: 10.1186/1471-2164-10-163 19379481 PMC2678161

[22] Lin R Maeda S Liu C Karin M Edgington TS. A Large Noncoding RNA is a Marker for Murine Hepatocellular Carcinomas and a Spectrum of Human Carcinomas. Oncogene (2007) 26(6):851–8. doi: 10.1038/sj.onc.1209846 16878148

[23] Schmidt LH Spieker T Koschmieder S Schaffers S Humberg J Jungen D. The Long Noncoding MALAT-1 RNA Indicates a Poor Prognosis in non-Small Cell Lung Cancer and Induces Migration and Tumor Growth. J Thorac Oncol (2011) 6(12):1984–92. doi: 10.1097/JTO.0b013e3182307eac 22088988

[24] Han Y Liu Y Nie L Gui Y Cai Z. Inducing Cell Proliferation Inhibition, Apoptosis, and Motility Reduction by Silencing Long Noncoding Ribonucleic Acid Metastasis-Associated Lung Adenocarcinoma Transcript 1 in Urothelial Carcinoma of the Bladder. Urology (2013) 81(1):209 e1–7. doi: 10.1016/j.urology.2012.08.044 23153939

[25] Tano K Mizuno R Okada T Rakwal R Shibato J Masuo Y. MALAT-1 Enhances Cell Motility of Lung Adenocarcinoma Cells by Influencing the Expression of Motility-Related Genes. FEBS Lett (2010) 584(22):4575–80. doi: 10.1016/j.febslet.2010.10.008 20937273

[26] Gutschner T Hammerle M Eissmann M Hsu J Kim Y Hung G. The Noncoding RNA MALAT1 is a Critical Regulator of the Metastasis Phenotype of Lung Cancer Cells. Cancer Res (2013) 73(3):1180–9. doi: 10.1158/0008-5472.CAN-12-2850 PMC3589741 23243023

[27] Tibshirani R. The Lasso Method for Variable Selection in the Cox Model. Stat Med (1997) 16(4):385–95. doi: 10.1002/(sici)1097-0258(19970228)16:4<385::aid-sim380>3.0.co;2-3 9044528

[28] Liu M Zhang H Li Y Wang R Li Y Zhang H. HOTAIR, a Long Noncoding RNA, Is a Marker of Abnormal Cell Cycle Regulation in Lung Cancer. Cancer Sci (2018) 109(9):2717–33. doi: 10.1111/cas.13745 PMC6125477 30047193

[29] Molina-Crespo A Cadete A Sarrio D Gamez-Chiachio M Martinez L Chao K. Intracellular Delivery of an Antibody Targeting Gasdermin-B Reduces HER2 Breast Cancer Aggressiveness. Clin Cancer Res (2019) 25(15):4846–58. doi: 10.1158/1078-0432.CCR-18-2381 31064780

[30] Croes L Beyens M Fransen E Ibrahim J Vanden Berghe W Suls A. Large-Scale Analysis of DFNA5 Methylation Reveals its Potential as Biomarker for Breast Cancer. Clin Epigenet (2018) 10:51. doi: 10.1186/s13148-018-0479-y PMC5896072 29682089

[31] Zhang X He X Liu Y Zhang H Chen H Guo S. MiR-101-3p Inhibits the Growth and Metastasis of Non-Small Cell Lung Cancer Through Blocking PI3K/AKT Signal Pathway by Targeting MALAT-1. BioMed Pharmacother (2017) 93:1065–73. doi: 10.1016/j.biopha.2017.07.005 28738500

[32] Liu J Tian W Zhang W Jia Y Yang X Wang Y. MicroRNA-142-3p/MALAT1 Inhibits Lung Cancer Progression Through Repressing Beta-Catenin Expression. BioMed Pharmacother (2019) 114:108847. doi: 10.1016/j.biopha.2019.108847 30970294

[33] Song J Sun Y Cao H Liu Z Xi L Dong C. A Novel Pyroptosis-Related lncRNA Signature for Prognostic Prediction in Patients With Lung Adenocarcinoma. Bioengineered (2021) 12(1):5932–49. doi: 10.1080/21655979.2021.1972078 PMC8806662 34488540

[34] Wang WJ Chen D Jiang MZ Xu B Li XW Chu Y. Downregulation of Gasdermin D Promotes Gastric Cancer Proliferation by Regulating Cell Cycle-Related Proteins. J Dig Dis (2018) 19(2):74–83. doi: 10.1111/1751-2980.12576 29314754

[35] Zhou Z He H Wang K Shi X Wang Y Su Y. Granzyme A From Cytotoxic Lymphocytes Cleaves GSDMB to Trigger Pyroptosis in Target Cells. Science (2020) 368(6494):eaaz7548. doi: 10.1126/science.aaz7548 32299851

