# Supplementary material for: Pyroptosis-Related LncRNA Signatures Correlate With Lung Adenocarcinoma Prognosis
Source: Front Oncol. 2022 Mar 2;12:850943. doi: 10.3389/fonc.2022.850943 (PMC8924059; doi:10.3389/fonc.2022.850943)
Supplement: Supplementary file 1 [file DataSheet_1.docx]

## Supplementary Figures


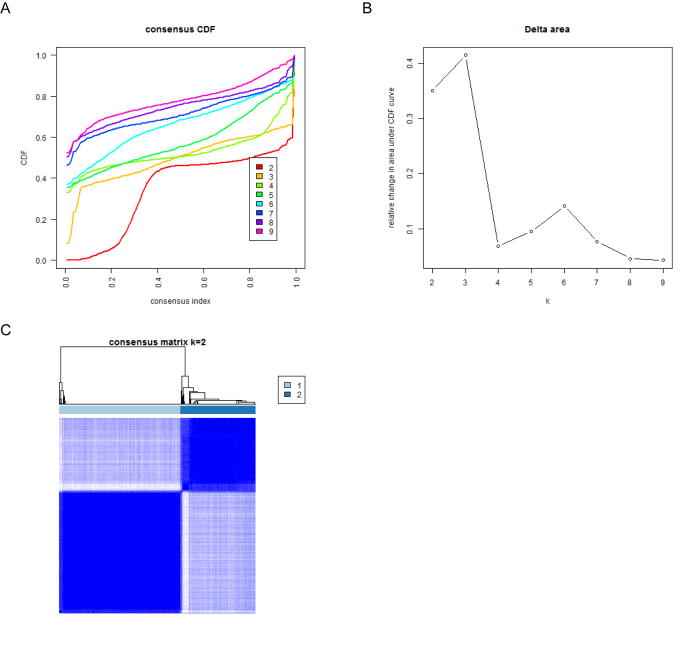


**Supplementary Figure S1.**Consensus clustering analysis based on the mRNA expression profiles of 52 pyroptosis-related genes. (**A, B**) Consensus clustering model with cumulative distribution function by k from 2 to 9. (**C**) Consensus matrix when k=2.


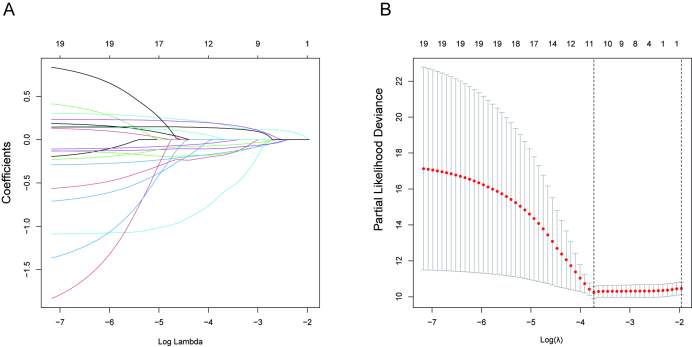


**Supplementary Figure S2.** LASSO Cox regression analysis was applied to model construction. **(A, B)** Screening the optimal parameter (lambda), which is represented by the vertical black line in the plot.
