# Supplementary material for: Pyroptosis-Related LncRNA Signatures Correlate With Lung Adenocarcinoma Prognosis
Source: Front Oncol. 2022 Mar 2;12:850943. doi: 10.3389/fonc.2022.850943 (PMC8924059; doi:10.3389/fonc.2022.850943)
Supplement: Supplementary file 2 [file Table_1.docx]

Supplementary Material

Supplementary Tables

Table S1. Summary of pyroptosis-related genes.

| Pyroptosis-related genes | | | | |
| --- | --- | --- | --- | --- |
| BAK1 | CHMP4C | IL1A | GPX4 | NLRP7 |
| BAX | CHMP6 | IL1B | GSDMA | NOD1 |
| CASP1 | CHMP7 | IRF1 | GSDMB | NOD2 |
| CASP3 | CYCS | IRF2 | GSDMC | PJVK |
| CASP4 | ELANE | TP53 | IL6 | PLCG1 |
| CASP5 | GSDMD | TP63 | NLRC4 | PRKACA |
| CHMP2A | GSDME | AIM2 | NLRP1 | PYCARD |
| CHMP2B | GZMB | CASP6 | NLRP2 | SCAF11 |
| CHMP3 | HMGB1 | CASP8 | NLRP3 | TIRAP |
| CHMP4A | IL18 | CASP9 | NLRP6 | TNF |
| CHMP4B | GZMA |  |  |  |

Table S2. Primers used in this study.

| Gene | Forward primer | Reverse primer |
| --- | --- | --- |
| AC004865.2 | CCTTTCGCACCAAGGACCC | GGCCCTGGAATCAGAGAAAGT |
| LINC02178 | ACAGCACGAGAGTTGTAGGC | CGGCCGAGGCTCTAATGAAA |
| AC004704.1 | TCATGTTTTTCCCGGGATGTCA | CTTTGTTCTCCCTCTGTTCTTGT |
| LINC02390 | TCAAAAGAACACTTGCCCGC | GGTCTCCACTGTAGAATACGCA |
| AC026355.2 | TGACTGCAGAGAAGTAGCGT | GTATGCCATCCACCACCACA |
| AC010999.2 | TAAGGCTCTCATTCTGGCTGC | TAAGGCTCTCATTCTGGCTGC |
| AC090559.1 | GAGATGCCCATGTCTAGGACC | ATCTTGGACGCTCTAGGGAC |
| AC024075.2 | TGACTTTGGCTGGGTCACTC | GGTTCCTAGCCAGCCTTCTT |
| AP005137.2 | TTCTCATGACTCCTGCGCTT | ACTGGACAGACTGCAAAGGG |
| AC026368.1 | CCCCTCTCCCCAGTTGTCTA | GCAAGGTGTGCCTATCAACG |
| AC012085.2 | AGCCCCTAGCTAAGCCTCTA | TGATTCTTGCCGTTGAGACA |

Table S3. The clinical characteristics of the TCGA cohorts.

| Clinical characteristic | | TCGA cohort |
| --- | --- | --- |
| Age | <65 | 205 |
|  | ≥65 | 244 |
|  | unknown | 5 |
| Gender | Male | 202 |
|  | Female | 247 |
|  | unknown | 5 |
| Stage | Ⅰ-Ⅱ | 350 |
|  | Ⅲ -Ⅳ | 99 |
|  | unknown | 5 |
